# Therapeutic Effects of a Newly Developed 3D Magnetic Finger Rehabilitation Device in Subacute Stroke Patients: A Pilot Study

**DOI:** 10.3390/brainsci12010113

**Published:** 2022-01-14

**Authors:** Sung-Hoon Kim, Dong-Min Ji, Chan-Yong Kim, Sung-Bok Choi, Min-Cheol Joo, Min-Su Kim

**Affiliations:** 1 Department of Electronics Convergence Engineering, Wonkwang University, 460 Iksandae-ro, Iksan 54538, Korea; kshoon@wku.ac.kr (S.-H.K.); anggole94@gmail.com (D.-M.J.); 2 Department of Rehabilitation Medicine, College of Medicine, Wonkwang University, 460 Iksandae-ro, Iksan 54538, Korea; cy7974@naver.com (C.-Y.K.); csb950801@naver.com (S.-B.C.); jmc77@hanmail.net (M.-C.J.)

**Keywords:** hand, finger, magnets, rehabilitation, robotics, stroke, upper extremity

## Abstract

We developed a magnetic-force-based three-dimensional (3D) rehabilitation device that can perform motor rehabilitation treatment for paralyzed fingers, regardless of upper extremity movement and position, and investigated the therapeutic effects of the device. An end-effector type rehabilitation device that can generate magnetic fields in three directions was developed using electromagnets and permanent magnetics. A double-blinded randomized controlled pilot study was conducted with a total of 12 patients. The intervention group had rehabilitation treatment using the developed magnetic finger rehabilitation device for 30 min a day for four weeks. The control group underwent exercise rehabilitation treatment. The control group received conventional occupational therapy on the upper limbs, including hands, from an occupational therapist, for the same amount of time. Adverse effects were monitored, and the patient’s sensory or proprioceptive deficits were examined before the intervention. No participants reported safety concerns while the intervention was conducted. The Wolf Motor Function Test (WMFT) scores were significantly improved in the intervention group (from 13.4 ± 3.6 to 20.9 ± 4.0 points) compared to the control group (from 13.1 ± 4.0 to 15.2 ± 3.8 points) (*p* = 0.016). The patients in the intervention group (from 88 ± 12 to 67 ± 13 s) showed greater improvement of WMFT times compared to the control group (from 89 ± 10 to 73 ± 11 s) (*p* = 0.042). The Manual Function Test and the upper limb score of the Fugl-Meyer Assessment were significantly improved in the intervention group compared with the control group (*p* = 0.038 and *p* = 0.042). The patients in the intervention group also showed significantly greater enhancement of the Korean version of the modified Barthel Index than the control group (*p* = 0.042). Rehabilitation treatment using the 3D magnetic-force-driven finger rehabilitation device helped improve finger motor function and activities of daily living in subacute stroke patients.

## 1. Introduction

Motor function impairment is a complication observed in approximately 80% of stroke patients and mainly involves a unilateral limb or the face [1]. Upper extremity motor impairments are difficult to recover from and increase dependence on others for daily life activities. In one study, approximately 20% of patients showed recovery of upper extremity motor function three months after stroke onset, and 50% of patients had upper extremity motor impairment four years after stroke onset [2]. The level of hand motor impairment is closely related to daily life activities and has a significant impact on quality of life [3]. Therefore, physiatrists have developed various treatment techniques to treat hand motor impairments caused by stroke [2]. However, as the human hand has highly complicated functions, restoring hand motor function remains an ongoing challenge in rehabilitation [3].

Recently, robotic therapy techniques have been actively applied to treat hand motor impairments in stroke patients [4,5,6]. Initially, exoskeleton robots made of metals were mainly used to compensate for muscle weakness caused by paralysis and to support hand movements [4,5,6]. Following advancements in material engineering, soft robots made of easily deformable materials such as liquids, gels, and polymers have been actively developed, and these soft robots have been improved such that they can perform movements that closely resemble those of human hands [7,8]. These soft hand robots mainly use air, cables, and hydraulic devices as the driving mechanism and often have wearable designs in the form of gloves [7,8]. Soft hand robots help to improve patient compliance [9,10], and the use of soft hand robots with conventional physical therapy has led to increased positive effects on the recovery of hand motor function [3,7,11].

Herein, we proposed a novel hand rehabilitation device using a magnetic force. We hypothesized that magnetic force could be effectively utilized in assisting with the power needed to move paralyzed arms and legs due to diseases such as stroke. Prior to this study, we conducted a study with healthy people to see if they could exercise their fingers using a magnetic force [12,13]. As a result, we identified the therapeutic potential to apply permanent electromagnets to patients’ finger rehabilitation exercises [12,13]. Little research has been conducted on magnetic-force-based rehabilitation robots or devices developed for hand rehabilitation treatment in stroke patients. Therefore, this study aimed to develop a 3D finger rehabilitation device that can perform finger exercises using magnetic forces. We investigated the therapeutic effects of this device combined with conventional rehabilitation therapy for subacute stroke patients with finger paralysis.

## 2. Materials and Methods

### 2.1. Development of the Device

The developed electromagnetic rehabilitation system with a multilink magnetic device on the finger provides flexion and extension of fingers because the applied AC magnetic field generates magnetic forces (attractive and repulsive forces) [12,13]. These forces induce flexing or extending motions of the fingers. The magnetic forces required to move the fingers as much as desired can be controlled by the amount of current flowing through the coil [12,13].

**Figure 1:**
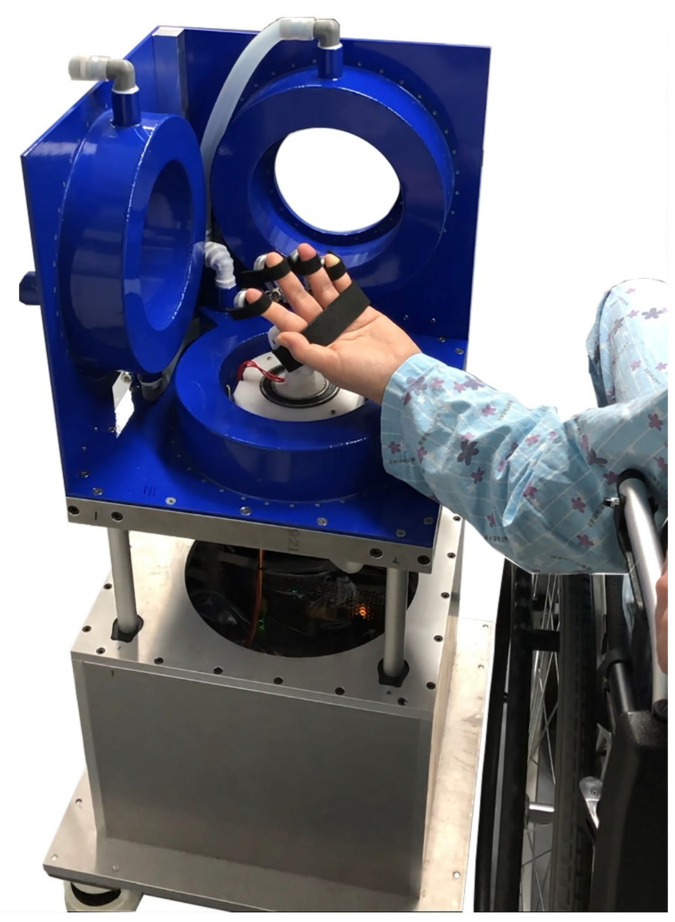
3D magnetic-force-driven finger rehabilitation device. The installed magnet-array device on the patient’s fingers generates magnetic attractive and repulsive forces by the driving magnetic field in the 3D coil system. Rehabilitation therapy can be performed by assisting the movement of the paralyzed fingers using these magnetic forces.

The proposed three-dimensional (3D) hand rehabilitation system with a magnetic multilink device can provide active flexion and extension of the finger, regardless of the hand position, because of real-time hand position sensing (Figure 1).

The vector (vector) sum of the three-dimensional electromagnetic system magnetic field can control the direction of the magnetic field. The magnetic field direction is adjusted according to the current level flowing to each coil. The current power available to each coil can be calculated through the rotational conversion matrix below.

$$\left[\begin{array}{c} Ix\\ Iy\\ Iz \end{array}\right]=I\sin\omega t\;\left[\begin{array}{ccc} \cos(\phi ) & -\sin(\phi ) & 0\\ \sin(\phi ) & \cos(\phi ) & 0\\ 0 & 0 & 1 \end{array}\right]\;\left[\begin{array}{ccc} \cos(\theta ) & 0 & \sin(\theta )\\ 0 & 1 & 0\\ -\sin(\theta ) & 0 & \cos(\theta ) \end{array}\right]\;\left[\begin{array}{c} 0\\ 0\\ 1 \end{array}\right]$$

*θ* is the Y-axis angle (Pitch), ψ is the Z-axis angle (Yaw), *Ix*, *Iy*, and *Iz* are electric currents that are edging to the X-axis, Y-axis, and Z-axis coils, respectively. The coil continuously changes the direction of the magnetic field by shedding the current of the sine waveform. In addition, magnetic torque occurs between the magnet and the magnetic field attached to the finger, which aids finger movement. The specifications of the three-axis coil are as shown in Table 1.

**Figure 2:**
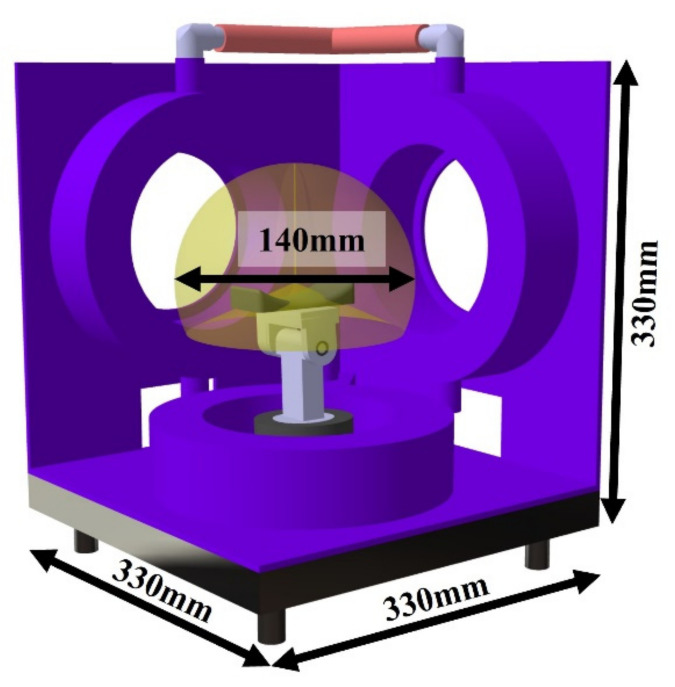
Size of 3D magnetic finger rehabilitation device.

The height of the finger rehabilitation area can be adjusted from 650 mm to 850 mm by the linear motor. As shown in Figure 2, the three-axis coil system is 330 mm wide and vertical. In addition, movement is possible in all areas inside the hemisphere range of 140 mm diameter in the central part.

**Figure 3:**
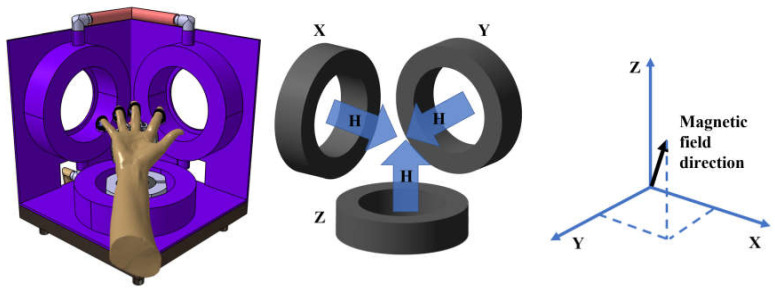
Direction of the magnetic force.

Two potentiometers were installed on the arm rest to detect the yaw and pitch angles of the hand rest [12,13]. Pitch and yaw angles are detected using potentiometers located at the joints of the hand base. Pitch and yaw angles can detect the direction of the palm. The system can ensure stable movement when the direction of the magnetic field matches the direction of the palm, as shown in Figure 3.

Therefore, yaw and pitch angle can only detect the palm direction; thus, two potentiometers were used. Suppose the position of the hand is changed. In that case, the change in angle would be fed back to the current controller of the coils, and the direction of the magnetic field is automatically changed to the hand’s position by the control algorithm. Therefore, a constant external force can be continuously applied for finger rehabilitation, regardless of the patient’s hand position.

### 2.2. Subjects

The participants in this study were subacute stroke patients within 3 months of onset who had an upper extremity strength of grade 3, including a paralyzed upper extremity, in the manual strength test. Patients who had a modified Tardieu Scale of grade 3 or higher and could not use the rehabilitation device due to spasticity or severe muscle shortening, those who had severe cognitive impairment and could not understand the instructions of the physical therapists, those who could not maintain sitting balance, and those who could not undergo adequate rehabilitation treatment due to serious medical conditions such as pneumonia were excluded from the study [14].

This was a parallel-group, double-blind, randomized controlled pilot trial with participants randomly allocated in a 1:1 ratio between treatment and placebo groups. Randomization was performed by an additional statistician prior to trial commencement using a block randomization process to ensure equal numbers in each treatment arm.

The participants were randomly assigned to the intervention and control groups. While patients in the control group received conventional occupational therapy for upper limb functional recovery, patients in the intervention group received rehabilitation treatment using the magnetic hand rehabilitation device. The conventional occupational therapy program consisted of the range of motion exercise of the upper limb, finger stretching, sensory stimulation, and strengthening exercises. The intervention was conducted for four weeks, for 30 min once a day, and the total amount of administered time was equal in both groups. In addition to this intervention, stroke rehabilitation programs such as neurodevelopmental therapy, muscle strengthening exercises, and gait training, which are generally administered to stroke patients, were performed for both groups, twice a day for an hour.

### 2.3. Magnetic Finger Rehabilitation Protocol

The intervention group underwent rehabilitation treatment using a magnetic finger rehabilitation device. Rehabilitation exercises using the device included the following exercises: (1) flexion/extension of fingers, (2) a sequential finger-thumb opposition exercise, and (3) twisting of the metacarpophalangeal joint. Flexion/extension of the fingers was performed using magnetic force to stimulate the proprioceptive sense of the hand and prevent shortening of finger muscles. The finger-thumb opposition exercise using the thumb and four other fingers was conducted to assist in the functional motions of a pinch grip. Twisting of the metacarpophalangeal joint was conducted to stretch the distal joint of the hand. All exercises were designed as active-assisted exercises, for the participants to perform as many movements as possible with the help of magnetic force. If the participants required more assistance due to shortening of muscles, the exercises were performed by controlling the magnetic force. Each exercise was conducted using the magnetic finger rehabilitation device for 10 min, with one treatment session lasting approximately 30 min.

### 2.4. Outcome Measures

The Wolf Motor Function Test (WMFT) was assessed as the primary outcome to compare the treatment effects between the intervention and control groups. The assessor evaluated the patient’s hand function without knowing the group to which the subject was assigned. The WMFT consisted of 15 functional tests and two muscle strength tests, involving complex movements from proximal to distal interphalangeal joints, which comprehensively evaluated upper extremity motor function [15]. Each of the 15 evaluation items measured the time required for the participants to completely perform the given tasks, and the maximum time allowed was 120 s. The WMFT score reflects the level of hand movement while performing the various tasks [16]. As previously described, the WMFT has high reliability and validity for the evaluation of patients with severe hand motor impairment [16]. The score can range from 0 to 75 points, and a higher score indicates superior hand motor function.

The Manual Function Test (MFT), upper limb movement evaluation items from the Fugl-Meyer Assessment (FMA_U), and the Korean version of the modified Barthel Index (K-MBI) were used to assess secondary outcomes. The MFT is a tool developed to evaluate upper limb motor function in patients with hemiplegia after stroke [17,18]. The MFT has high reliability and validity for the evaluation of upper limb motor function in stroke patients with severe hemiplegia [18]. The score can range from 0 to 100 points, with higher scores indicating better hand motor function. The FMA_U is a widely used tool to evaluate sensorimotor impairment in stroke patients [19]. Each item was evaluated on a three-point scale, and the maximum possible score was 66 points [19].

K-MBI is an evaluation tool for assessing the independence of activities of daily living. K-MBI consists of 10 evaluation items (personal hygiene, bathing, eating, toileting, stair climbing, dressing, defecation, voiding, walking, chair-bed transfer) [20]. The score of each item is divided into five phases by item, and nine weights are applied depending on the proportion of the content [20]. The total score is 100 points, and the higher the score, the more independent the patient can be in their daily lives.

Both primary and secondary outcomes were evaluated twice: before the treatment and after four weeks of treatment. Basic information such as age, sex, stroke type, dominant hand, affected side, period from onset to treatment, spasticity severity, National Institutes of Health Stroke Scale (NIHSS) scores, and scores on the Montreal Cognitive Assessment (MoCA) were obtained before initiation of treatment.

### 2.5. Statistics

To determine differences in the baseline parameters between the two groups, the Mann–Whitney U-test for continuous variables and Fisher’s exact test for categorical variables were used. The changes of each variable from before to after the treatment within groups was analyzed by Wilcoxon signed-rank tests. Mann–Whitney U tests were performed to compare the therapeutic effects between groups. To satisfy an α level of 0.05 with a power of 0.80 in an actual randomized controlled trial, at least 14 subjects were required in each of the two groups [21]. *P*-values below 0.05 were defined as statistically significant, and all statistical analyses were performed using SPSS 22.0 (SPSS Inc., Chicago, IL, USA).

## 3. Results

### 3.1. Safety Concerns

Physiatrists, occupational therapists, and robotic development engineers monitored the probable side effects while the patients performed rehabilitation therapy using the magnetic-force-driven rehabilitation device. Prior to the beginning of the intervention, the patient’s sensory or proprioceptive deficits were examined. In addition, the physiatrist confirmed whether the patients had soft tissue injury or musculoskeletal pain on the upper limb, before and after each intervention session. No participants experienced safety concerns while the intervention was conducted.

### 3.2. Demographic Characteristics

A total of 12 participants were recruited, with six participants in each of the intervention and control groups. The mean age of the participants was 60.5 ± 5.4 years, and the mean period from stroke onset to treatment was 32.2 ± 5.2 days. There were no significant differences in the demographic and clinical characteristics between the two study groups (Table 2). Additionally, the NIHSS and MoCA scores, spasticity severity, and sequelae were not significantly different between the intervention and control groups.

### 3.3. Primary Outcome Measures

Baseline scores for the WMFT, MFT, FMA_U, and K-MBI were not significantly different between the intervention and control groups. After four weeks of treatment, WMFT scores significantly increased from 13.4 ± 3.6 points to 20.9 ± 4.0 points in the intervention group (*p* < 0.001) and from 13.1 ± 4.0 points to 15.2 ± 3.8 points in the control group (*p* = 0.008) (Table 3). WMFT times significantly decreased from 88 ± 12 s to 67 ± 13 s in the intervention group (*p* < 0.001) and from 89 ± 10 s to 73 ± 11 s in the control group (*p* = 0.004). The WMFT scores were significantly improved to a greater extent in the intervention group than in the control group (*p* = 0.016), and the WMFT time significantly decreased to a greater extent in the intervention group than in the control group (*p* = 0.042).

### 3.4. Secondary Outcome Measures

After four weeks of treatment, MFT scores significantly increased from 22.5 ± 2.9 points to 39.3 ± 3.5 points in the intervention group (*p* < 0.001) and from 23.1 ± 3.1 points to 31.7 ± 3.4 points in the control group (*p* = 0.012). However, the intervention group showed a significantly better MFT score than the control group (*p* = 0.038). FMA_U scores, which reflect upper limb motor function, significantly increased from 23.8 ± 2.9 points to 33.0 ± 3.4 points in the intervention group (*p* = 0.002) and from 22.9 ± 2.5 points to 26.8 ± 3.0 points in the control group (*p* = 0.034). The intervention group showed a significantly better FMA_U score than the control group (*p* = 0.042). K-MBI scores were significantly improved in both groups, from 46 ± 7 points to 68 ± 10 points in the intervention group (*p* < 0.001) and 47 ± 8 points to 60 ± 10 points in the control group (*p* = 0.004). The intervention group showed a significantly better K-MBI score than the control group (*p* = 0.042).

## 4. Discussion

Stroke patients who underwent four weeks of rehabilitation treatment using a magnetic finger rehabilitation device showed greater recovery of hand motor function than patients who underwent only conventional rehabilitation treatment. The recovery of impaired hand motor function contributed to improved performance of and independence in daily activities in the stroke patients.

The magnetic finger rehabilitation device can be used to perform rehabilitation treatment by inducing finger movements based on the magnitude and direction of the magnetic field between the electromagnetic field generated from the driving coil and the permanent magnet attached to the finger without using mechanical parts such as motors. The magnitude and direction of the magnetic force must be appropriately controlled to implement various finger motions, such as bending, extension, and twisting of fingers. We conducted a preliminary experimental study with healthy adults to develop a system that can control the magnetic force required for rehabilitation treatment based on the level of hand motor function, and the device that was developed was subsequently used in this study [13].

The magnetic finger rehabilitation device has several advantages for conducting rehabilitation treatment. The magnetic rehabilitation device uses a simple mechanism to induce movements of affected hands with muscle paralysis using a coil and a permanent magnet. This allows easy manufacture and a small size of the device. It also has the advantage that applying this device to rehabilitation therapy is relatively simple. Once the patients wore the ring form magnets on their fingers and then put the hand into the device, finger movement could be induced. We assumed that the convenience helped patients participate cooperatively in rehabilitation therapy using this device. In addition, various movements can be exercised, and patients can use their maximum remaining muscle strength during exercises such as flexion and extension of fingers. Conventional robots have a fixed axis, which limits the diversity of treatment methods available to patients and physical therapists. The magnetic rehabilitation device simply requires the user to match the desired movement to the direction of the magnetic force and allows various hand movement exercises, such as simple adduction, twisting, and touching with either hand, in addition to simple grasping and stretching movements.

Safety assessment is a vital aspect of the rehabilitation device development process. There are no uniform guidelines for the safety validation of devices or robots closely interacting with humans based on safety skills and validation protocols [22]. Establishing safety guidelines is a great challenge for rehabilitation robotic researchers, since the variety of patient pathologies that can affect pain perception or cause movement restrictions should also be considered [23]. Therefore, the researchers addressed robot safety by monitoring adverse event occurrence currently [23]. The common adverse events were known as soft-tissue-related adverse events and musculoskeletal adverse events [22]. Soft tissue-related adverse events in stationary gait trainers included skin irritation, skin reddening, skin abrasions, and open skin lesions and bruising, as well as discomfort and pain to soft tissue areas [24]. Musculoskeletal adverse effects extracted from the systematic review were tendinopathy, a tibia fracture, muscle pain, lower back pain, malleolus pain and discomfort, and pain to joints [24]. We continued to monitor the patients’ status during the intervention, because magnetic-force-driven rehabilitation devices may have similar side effects. We immediately pressed the emergency stop button if a malfunction or abnormality was detected. If the patients felt pain or unwanted force on the finger joints, they were educated to immediately move their hands away from the magnetic field. It is assumed that these points may have contributed to preventing the injury of patients during this study, and the authors believe that subsequent devices should be developed with safety in mind.

Herein, magnetic-force-based hand rehabilitation treatment with conventional stroke exercise treatment further improved hand motor function and daily activities compared to conventional exercise treatment alone. We assumed that similar mechanisms revealed from the effect of rehabilitation robotics for stroke patients contributed to the recovery of hand function through the magnetic-force-driven rehabilitation device. For example, the magnetic finger rehabilitation device can repeatedly perform exercise therapy for a set period of time without additional help from physical therapists [11]. The device allows intensive and repetitive rehabilitation treatment patterns to be performed, which would have effectively provided motor learning to the patients [10]. Moreover, the patients underwent a variety of finger exercises, including flexion and extension, by using magnetic force as an auxiliary force based on the level of muscle strength. The patients with reduced sensation of proprioception could also visually check their level of movement. Such effects could have provided psychological stability and motivation for the patients to undergo treatment [25].

We observed that WMFT, MFT, and FMA_U scores were significantly improved in the intervention group compared with the control group; however, K-MBI scores were not significantly different between the two groups after the intervention. This may have been due to the nature of the K-MBI, which evaluates overall independence in daily life, unlike the WMFT and MFT, which evaluate hand function, and the FMA_U, which evaluates hand motor function. Although motor function of the hand recovers to a large extent after stroke, minor impairments continue to affect other aspects of function, such as gait, control over urination and defecation, and eating, which are required for independent daily life [26]. However, K-MBI scores were significantly improved after the treatment in both groups, suggesting that the magnetic finger rehabilitation device does not have negative effects on improvements in independence in daily life. Therefore, patients undergoing stroke rehabilitation should receive exercise and occupational therapy in addition to hand rehabilitation using a magnetic device, and the device may be helpful in improving hand function.

Unlike the early forms of other rehabilitation robots that have been developed, the magnetic finger rehabilitation device used in this study does not treat patients on behalf of physical therapists. However, the therapeutic effects of this device on stroke patients are expected to reduce the burden for physical therapists and provide more diverse treatment options when automated robotization is available in the future. Currently, the device in this initial form requires more time and effort to prepare and perform the intervention treatment than physical therapists who could directly perform the treatment. However, the findings of this study show that magnetic force may be used to replace existing devices, such as motors and wires, and can become an effective treatment technique in the near future.

Several limitations need to be considered in the interpretation of the findings of this study. The twisting motion was adopted as one of the treatment protocols for the proposed device. However, it is not known if this twisting motion has a therapeutic effect on finger rehabilitation exercises. This device was exceptional at generating this unique movement. Future studies are required to address what kind of mechanisms play a positive role in the twisting motion of the fingers.

## 5. Conclusions

We developed a prototype device of a 3D finger rehabilitation system that can induce desired movements using a magnetic field, regardless of the position of the upper extremity. The developed magnetic finger rehabilitation device can facilitate various finger movements, such as flexion and extension, pinching motions of the thumb and the little finger, and twisting. The device allows the user to perform passive joint motions and auxiliary active motions without side effects. Conventional treatment combined with hand rehabilitation treatment using this device further improved the hand motor function of patients compared to conventional treatment alone. Therefore, this 3D magnetic-force-based hand rehabilitation device may be useful in restoring hand motor function in patients with various brain diseases and neurological disorders that cause muscle paralysis.

## Figures and Tables

**Table 1:** Specifications of the three-axis coil.

	Radius (mm)	Wire Diameter (mm)	Resistance (Ω)	Number of Turns
3-axis coil	115	2.0	1.65	405

**Table 2:** Baseline characteristics of the participants.

Patient No.	Group	Age (y)	Gender	Type	Affected Side	Lesion Location	Dominant Hand	Period after Onset (Days)	NIHSS	MoCA	Spasticity (MTS) †
1	Intervention	56	M	Infarct	Right	BG	Right	35	11	21	1
2	Intervention	62	F	Hemorrhage	Left	BG	Right	37	9	20	1
3	Intervention	63	F	Infarct	Right	MCA	Right	31	8	19	0
4	Intervention	59	M	Infarct	Left	MCA	Right	29	12	22	0
5	Intervention	60	M	Infarct	Right	IC	Right	33	11	23	0
6	Intervention	61	F	Infarct	Left	IC	Right	32	11	24	0
7	Control	63	F	Infarct	Left	MCA	Right	35	8	22	0
8	Control	63	M	Infarct	Right	MCA	Right	36	8	19	1
9	Control	61	M	Hemorrhage	Right	BG	Right	30	13	18	0
10	Control	60	F	Hemorrhage	Right	BG	Right	27	12	24	1
11	Control	58	M	Infarct	Left	IC	Right	28	11	24	0
12	Control	60	F	Infarct	Left	IC	Right	33	10	23	0

NIHSS, National Institute of Health Stroke Scale; MoCA, Montreal Cognitive Assessment; MTS, modified Tardieu Scale; BG, Basal ganglia; MCA, Middle cerebral artery; IC, Internal capsule. †: Elbow, wrist, and finger flexor muscles of affected side were evaluated. If even one muscle showed spasticity, it was determined positive.

**Table 3:** Comparison of the outcome measures between the intervention group and the control group.

	Intervention Group			Control Group			MW-U	*p*–Value †
	Pre	Post	Δ Post-Pre	Pre	Post	Δ Post-Pre		
WMFT score	13.4 (13.1)	20.9 (19.5)	7.5	13.1 (12.9)	15.2 (14.8)	2.1	3.500	0.016 *
WMFT time (sec)	88 (84)	67 (65)	21	89 (85)	73 (71)	16	5.500	0.042 *
MFT	22.5 (20.9)	39.3 (38.6)	16.8	23.1 (21.3)	31.7 (29.6)	8.6	4.500	0.038 *
FMA_U	23.8 (22.8)	33.0 (32.1)	9.2	22.9 (21.1)	26.8 (25.2)	4.1	5.500	0.042 *
K-MBI	46 (43)	68 (66)	22	47 (44)	60 (58)	14	5.500	0.042 *

Data are expressed as median and interquartile range (IQR). MW-U, Mann–Whitney U; WMFT, Wolf Motor Function Test; MFT, Manual Function Test; FMA_U, Upper limb score of the Fugl-Meyer Assessment; K-MBI, Korean version of the modified Barthel Index. * *p* < 0.05; †: analyzed by Mann–Whitney U-test for comparison of two groups after treatment.

## Data Availability

The datasets analyzed during the current study are available from the corresponding author on reasonable request.
